# The effect of food insecurity and stress on delay discounting across families: a COVID-19 natural experiment

**DOI:** 10.1186/s12889-022-13969-1

**Published:** 2022-08-19

**Authors:** Amanda K. Crandall, Nayana Madhudi, Bernadette Osborne, Autum Carter, Aliaya K. Williams, Jennifer L. Temple

**Affiliations:** 1 Department of Community Health and Health Behavior, University at Buffalo School of Public Health and Health Professions, Buffalo, NY USA; 2 Department of Exercise and Nutrition Sciences, University at Buffalo School of Public Health and Health Professions, Buffalo, NY USA

**Keywords:** Delay discounting, Stress, Food insecurity, Income, COVID-19, Families, Children, Adolescents

## Abstract

**Background:**

Delay Discounting is the extent to which one prioritizes smaller immediate rewards over larger, delayed rewards. The ability to prospect into the future is associated with better health decision-making, which suggests that delay discounting is an important intervention target for the prevention and treatment of chronic disease. Delay discounting decreases throughout development and stressful experiences, particularly those that accompany poverty, may influence this developmental trajectory. The current study leveraged the COVID-19 pandemic and resulting economic downturn as a natural experiment to understand how changes in food insecurity and psychological stress may associated with changes in delay discounting among parents, adolescents, and children.

**Methods:**

A stratified cohort of families (*N* = 76 dyads), established prior to the initial pandemic lockdowns, were asked to complete a follow-up survey in the summer of 2020, during reopening. Thirty-seven (49%) families had an older adolescent (aged 15 – 18 years) in the study and 39 (51%) had an elementary aged child (aged 7 – 12 years) in the follow-up study. Both data collection points included measurements of economic position, psychological stress, food security status, and delay discounting.

**Results:**

The results showed that pandemic food insecurity was associated with greater stress among parents (*β* = 2.22, *t*(65.48) = 2.81, *p* = 0.007). Parents, Adolescents, and children significantly differed in their response to psychological stress during the pandemic (*β* = -0.03, *t*(102.45) = -2.58, *p* = 0.011), which was driven by a trend for children to show greater delay discounting associated with an increase in psychological stress during the pandemic (*β* = -0.01, *p* = 0.071), while adolescents and parents showed no change.

**Conclusions:**

These findings add to the evidence that food insecurity is uniquely stressful among parents with no effects on delay discounting. Despite this, we found no evidence that food insecurity was stressful for child or adolescents. A trend in our data suggested that childhood, as compared with adolescence, may be an important developmental period for the association between stress and delay discounting. Future research should continue the longitudinal investigation of childhood stress and the developmental trajectory of delay discounting to ascertain how these effects may persist in adulthood.

**Supplementary Information:**

The online version contains supplementary material available at 10.1186/s12889-022-13969-1.

## Background

The prevention of chronic diseases, such as heart disease and diabetes, is of paramount importance in public health today [1]. However, despite the strong evidence for lifestyle approaches, such as adopting a more nutritious diet that successfully prevents such diseases [2], a large portion of the US population does not meet the current dietary or physical activity guidelines [3, 4], particularly if they are living in poverty [5]. One explanation is that many people place greater value on immediate rewards and discount future rewards, a process known as delay discounting (DD) [6–8]. Adults with greater DD tend to consume more calories in a laboratory eating task [9], have a greater risk for obesity [10], have more difficulty losing weight [11], and are more likely to have Prediabetes [12]. Although individual differences in DD have genetic origins [13, 14], environmental stimuli have been shown to impact state-level DD as well as the developmental trajectory of DD in children and adolescents.

As the frontal lobe develops throughout childhood and adolescence, DD decreases on average, meaning that adults display less DD than adolescents and adolescents less than children [15–17]. Evidence suggests that experiences with poverty in childhood may influence the developmental trajectory of DD. Both lower socioeconomic status (SES) and greater food insecurity, a household-level condition of limited or uncertain access to adequate food, which at the most severe levels is accompanied by hunger [18, 19], are associated with greater DD in both children and adults [20–22] Discounting the future may be adaptive in scarce environments because acquisition of the immediate reward is often more advantageous than planning for an uncertain future [23]. For example, experimental evidence shows that DD increases after adults read a narrative describing a financial crisis [24], suggesting that when future financial stability is in danger, immediate rewards are prioritized. Less is known about the effects of food insecurity specifically on the development of DD in children. Given that self-control develops slowly throughout childhood and adolescence [17, 25, 26] bouts of food insecurity could influence that development, but more time series studies are needed to fully understand these associations.

Psychological stress is another potential pathway by which experiences of poverty may impact DD, both in the moment of stress and during development. Neurobiologists have observed that after acute stress, there is less activity in the ventromedial prefrontal cortex, which is responsible for self-control [27]. Poverty and psychological stress are positively associated with one another [28, 29], and poverty increases the risk of exposure to stressful life events in adulthood [30] and adverse childhood experiences [31, 32]. Likewise, food insecurity is thought to be quite stressful for adults [33, 34] and developing youth [35]. Acute stress increases DD among adults [36, 37] and is associated with DD among children [37]. Longitudinally, greater exposure to stressful events lowers self-regulatory ability in early adolescence [38]. Retrospectively reported childhood stressors, including maltreatment and overall adversity, are associated with greater DD and general impulsivity in adulthood [39, 40]. Further, longitudinal evidence has shown that children who experience chronic stress show greater behavioral impulsivity as they age [40]. Although psychological stress and DD are associated in multiple studies, stress and poverty are often confounded with one another and more work is needed to understand the contributions of each to DD, particularly among developing youth.

The onset of the 2020 global pandemic of coronavirus disease 19 (COVID-19), caused by the severe acute respiratory syndrome coronavirus 2, brought with it varying levels of government response throughout the United States and all over the world [41, 42]. Government restrictions, along with the self-imposed precautions among consumers, caused one of the largest and fastest economic downturns in US history [41]. However, this economic downturn did not affect every sector of society in the same way. Many workers moved to working from home and had little change to their income [43]. Essential workers, such as delivery service providers and many healthcare workers, experienced a massive surge in work demand and overtime, likely increasing their income but also greatly increasing their stress levels [44]. Still other industries, such as hair stylists and fitness center employees, experienced a large drop in demand, were forced to close, and/or had hours cut or were laid off [45]. Due to this economic downturn, rates of poverty and food insecurity increased [42, 46], particularly among households with children [47]. The effects of these rapid changes on health and well-being are still under investigation, but evidence has suggested that the pandemic caused a high degree of psychological stress across the United States [48].

The evidence that is available suggests that food insecurity may narrow the temporal window for individuals, such that immediate rewards are much more salient [21, 22, 49]. Although there are associations among food insecurity, stress, and greater DD, these studies come from a disparate literature, with few longitudinal or experimental studies, and fewer still that have examined childhood and adolescence. The COVID-19 pandemic and resulting economic downturn have provided a natural experiment in the effects of a rapid lifestyle and economic shift. The current study sought to investigate how changes in food insecurity and stress related to the COVID-19 pandemic may be associated with one another and affect DD in adults, adolescents, and children. To do this, we reassessed a cohort of families who were originally studied prior to the onset of the pandemic. In our pre-pandemic study of this cohort, we showed that food insecurity was associated with greater DD across all age groups [49] and that parents with food insecurity had an acute rise in cortisol (a biological marker for acute stress) when they lost money in a laboratory experiment, but this stress response had no effect on DD [20]. The previous study also showed adolescents were more similar to their parents in terms of DD than were children [49]. In this follow-up study we aimed to use the COVID-19 pandemic as a natural experiment and see how changes in food insecurity and stress may be associated with changes in DD in this cohort. Based on the established developmental trajectory of DD [15–17], we first hypothesized that adolescents and children who had a longer period between assessments would have a larger decrease in DD compared with those who had a smaller period between assessments. Our previous results with this cohort suggested that acute stress has little effect on DD, potentially because food insecurity, and its associated chronic stress, have a larger and more chronic impact on DD [20]; We therefore hypothesized that a worsening of food insecurity and psychological stress would co-occur and would be associated with an increase in DD among adults. Finally, based on our previous results, and those that have shown adolescents to be more sensitive to food insecurity than children [50], we further hypothesized that adolescents would mirror their parents in these associations while children would not display any change beyond those expected between timepoints.

## Methods

### Study participants

The current analysis is a follow-up of a pre-pandemic cohort, recruited to study behavioral differences in families with and without food insecurity [49] and the role of stress in those differences [20]. This follow-up was not planned as part of the original study aims, but we saw an opportunity to reassess families, as the original measures were likely to be impacted by the pandemic. The participants in the original study were recruited from the community, based on responses to a screening survey. Participants were recruited based on receipt of food assistance (e.g., food bank donations, Supplemental Nutrition Assistance Program, etc.). This resulted in 106 families, 53 of whom reported receiving food assistance. To be eligible for the original study the parent needed to be over 18 years of age with an offspring who fell within the age range of 7 to 10 years old or 15 to 17 years old at the time of consent. No siblings were enrolled in this study and the children and adolescents were from different households. Each group had 27 parent–child dyads and 27 parent-adolescent dyads, with equal representation of male and female offspring. The sample was also counterbalanced by race/ethnicity, with at least 35% of the food assistance group reporting white race and at least 35% of the group without food assistance reporting non-white race or Hispanic ethnicity. Data collection for the original study spanned November 2018 through February 2020 and concluded before any lockdown measures were enacted in the Western New York area.

For the follow-up study, parents were emailed an invitation to participate in a “COVID-19 follow-up survey” in June 2020. Surveys were collected through September 2020. During this period, Western New York was moving through phase 3 (reopening with masking, testing, and reduced capacity of personal care businesses, and restaurants, as well as gatherings of 25 people or fewer) and phase 4 (reopening of higher education institutions, media production companies, and low risk entertainment services, such as museums, zoos, and botanical gardens as well as allowing gatherings of 50 people or less) of their staggered reopening plan. Families were emailed first and then contacted via phone for the opportunity to participate. We aimed to recruit at least 70% of the original cohort (*N* = 73 families) and to maintain the distribution described above in terms of offspring age group, sex, and race/ethnicity. Inclusion criteria were completion of all three appointments in the original study and that the same parent/guardian and offspring who completed the original study be the participants in the follow-up survey. There were no age limits placed on the follow-up sample.

### Procedures

Study procedures for both the original study and the follow-up survey were approved by the University at Buffalo Institutional Review Board. Procedures for the original study have been described elsewhere [20, 49]. Briefly, in the original study, families attended three separate appointments and were covertly exposed to a manipulation of small financial gains and losses, followed by a series of behavioral tasks, including the DD task. At the end of each of these appointments, participants completed surveys, including demographics (parents only), food insecurity, and the perceived stress scale (described below). The manipulation entailed asking the participants to play three different altered version of the Iowa Gambling Task [51], each of which was rigged to create a financial loss, gain, or neutral outcome. At the end of the experiment, the total winnings were summed, and the participants were paid this amount in cash in addition to their payment for participating. At the end of the final appointment, participants were debriefed about the research questions and the covert manipulation and were given the opportunity to remove their data if they wished, which none did [20, 49].

For the follow-up study, Parents consented for both themselves and, separately, for their offspring. After the parent provided consent for the offspring, a link to the adolescent/child survey was emailed directly to the offspring or to the parent if this option was selected by the parent. Therefore, both parent and offspring received a unique.url to access their surveys. The offspring was presented with an age-appropriate assent form and was informed that they would be able to withdraw from the follow-up study at any time without penalty. This study made use of remote consent, via REDCap [52], in which the participants signed via a computer screen using a mouse or a finger in a provided box to agree to the online consent process before providing any other information. Researchers were available via email and phone to answer any questions. If a parent or offspring did not provide consent/assent, they could not access the study questionnaires and simply exited the survey.

Following consent, the rest of the follow-up consisted of an online survey, also administered through REDCap [52]. Once participants accessed the surveys, they proceeded through them independently to answer each question. For all questions, participants had the option of selecting “Do not know” or “Prefer not to answer.” Participants were allowed to stop the survey and start again. If a survey was left incomplete, reminder calls and emails were sent to encourage completion. Participants were not made aware of the purpose of the study or research questions, however, in contrast to the original study, the follow-up survey did not have any manipulations.

For the parent surveys, they were first asked to complete a DD task (described below). Next, they answered a series of questions regarding their demographic information. This was followed by the United States Department of Agriculture (USDA) household food insecurity questionnaire, which was presented in the form of multiple-choice questions. Finally, they completed the Perceived Stress Scale. The offspring surveys were presented in a similar order to the parent survey without the demographic questionnaire. Attention check questions were scattered throughout the surveys. After both the parent and child completed their surveys, participants were mailed compensation in the form of $15 dollar gift cards of their choice.

### Assessments

#### Demographics and pandemic changes

In both the original study and the follow-up survey, participants completed the MacArthur SES questionnaire, which consists of questions regarding parent education level, household income, total assets, and total debts. Parents/guardians reported total household income, including employment income, government assistance, child support/alimony, and disability. This question offered ranges of income levels from “Less than $5,000” to “Over $100,000” (Table 1). Parents also reported the number of people living in the household, which was used to calculate income per person [53]. The follow-up survey included the same questions as the original survey, but with the addition of “during the COVID-19 pandemic” for the timeframe of each question.

**Table 1:** Participant characteristics between baseline and follow-up

Variable	Adolescent (*N* = 53) N (%)/Mean (SD)	Children (*N* = 53) N (%)/Mean (SD)	p	Adolescent (*N* = 37) N (%)/Mean (SD)	Children (*N* = 39) N (%)/Mean (SD)	p
Offspring Sex, n (%)			0.56			0.29
Female	27 (50.90)	30 (56.60)		20 (54.05)	15 (38.46)	
Male	26 (49.10)	23 (43.40)		17 (45.95)	24 (61.54)	
Offspring Race/Ethnicity, n (%)			0.62			0.56
Black/African American	11 (20.80)	14 (26.40)		7 (18.42)	11 (28.21)	
White	31 (58.50)	32 (60.40)		22 (57.89)	22 (56.41)	
Other or More than one race	11 (20.80)	7 (13.20)		9 (23.68)	6 (15.38)	
Hispanic or Latinx	10 (18.90)	4 (7.50)	0.09	6 (15.79)	3 (7.69)	0.23
Offspring Age, mean (SD)	16.12 (0.83)	9.20 (1.14)	0.00	17.06 (0.87)	10.33 (1.13)	0.00
Parent Sex, n (%)			0.04			0.06
Female	49 (92.50)	53 (100.00)		34 (89.47)	39 (100)	
Male	4 (7.50)	0 (0.00)		4 (10.53)	0 (0)	
Parent Race, n (%)			0.93			0.92
Black/African American	13 (24.50)	13 (24.50)		9 (23.68)	12 (30.77)	
White	36 (67.90)	36 (67.90)		27 (71.05)	25 (64.1)	
Other or More than one race	4 (7.60)	4 (7.60)		2 (5.26)	2 (5.12)	
Household Poverty, n (%)			0.92			0.51
Above the poverty line	36 (67.90)	32 (60.40)		20 (54.05)	22 (56.41)	
At/Below the poverty line	13 (24.50)	11 (20.80)		17 (45.95)	17 (43.59)	
Missing	4 (7.50)	10 (18.90)		0 (0)	0 (0)	
Parent Educational Attainment, n (%)			0.16			0.07
High school diploma or less	15 (28.30)	6 (11.30)		11 (29.73)	3 (7.69)	
Certificate or Associates Degree	15 (28.30)	12 (22.70)		12 (32.43)	9 (23.07)	
Bachelor’s Degree	12 (22.60)	20 (37.70)		6 (16.22)	17 (43.59)	
Master’s Degree or greater	10 (18.90)	12 (22.60)		9 (23.68)	8 (20.51)	
Missing	1 (1.90)	1 (1.90)		0 (0)	1 (2.56)	
Household Food Insecurity, n (%)			0.22			0.63
Full Food Security	35 (66.00)	35 (66.00)		26 (70.27)	26 (66.67)	
Marginal Food Security	8 (15.10)	4 (7.50)		6 (16.22)	4 (10.26)	
Low or Very Low Food Security	9 (17.00)	13 (24.60)		5 (13.51)	9 (23.07)	

#### Food insecurity

In both the original study and the follow-up survey, parents answered questions about their own feelings of food insecurity as well as the overall household food insecurity levels using the USDA 18-item household food security scale [54]. For example, questions included, “I/We worried whether our food would run out before I/we got money to buy more,” and “I/We relied on only a few kinds of low-cost food to feed the child because there wasn’t enough money for food.” Responses could be, “Often true, sometimes true, never true, I don’t know, or prefer not to answer.” Affirmative answers were summed and then broken into the standard categories of household food security (0 = Food secure, 1–2 = Marginal food security, 3–7 = Low food security, > 7 = Very low food security), with anyone falling into the “low” or “very low” categories being considered food insecure in the below analyses. The household food security scale is a valid measure of both population and individual level food insecurity [55]. In the original study, the timeframe for each question was, “in the last 12 months” and in the follow-up survey, the timeframe was “during the COVID-19 pandemic.”

#### Perceived life stress

In both surveys, all participants, including adolescents and children, were asked to complete the 10-item version of the Perceived Stress Scale in order to assess general life stress over the last month [56, 57]. This scale is commonly used to assess ongoing life stress and asks questions such as “How often have you felt that you were unable to control the important things in your life?” Participants answered on a Likert-Type scale anchored at 0 – Never and 4 – Very often [58]. Participants were able to refuse any questions throughout the study but needed to have answered at least eight items for their answers to be included in the analysis. To accommodate these missing values, scores were averaged and then multiplied by 10 to create scores that were comparable to previous research. The follow-up survey matched the original survey, but also included the same timeframe as above (i.e., “during the COVID-19 pandemic).

#### Delay discounting

DD was assessed using an adjusting amount DD task [59] presented using Inquisit (Millisecond, Seattle, WA) in the original study and via REDCap [52] for the follow-up survey. The DD task required participants to make choices between an amount of money available immediately or a larger amount of money available later. For example, questions included, “would you prefer $50 now or $1000 in one year?” and “Would you prefer $500 now or $1000 in one month?” The immediate value was adjusted until it was subjectively equivalent to the later, larger amount, the value of which is known as the indifference point. Indifference points were obtained at six delays (length of delay would progressively increase). The resulting individual score was calculated using area under the curve of the indifference points across delays, ranging from 0 to 1. A DD score of 1 would be achieved by always picking the larger, later amount and never discounting the future. Conversely a DD score of 0 would be achieved if a participant always chose the immediate amount, discounting the future no matter how long the delay or large the monetary reward. Therefore, when DD is scored using area under the curve, the resulting value is reverse scored, with higher scores indicating less DD (Please find a visual example in supplementary materials). This method has been validated against real-world monetary choices over delayed time periods [59].

Between age groups, the delays were consistent and ranged from one day to five years. However, the delayed amount was customized to the age group to capture discounting across the developmental spectrum. The delayed amounts were $1000 for parents, $100 for adolescents, and $50 for children. Indifference points across delays were checked for nonsystematic responses, and these points were removed if any were greater than the preceding indifference point by 20% of the delayed amount or more [60]. Except for the different presentation modalities noted above, this task was identical between the original study and the follow-up survey. Although the manipulation had no effect on DD in any group in the original study [49], only delay discounting data from the control visit (neutral outcome, no financial losses, or gains) in the original study has been included for a baseline measure in this analysis. The other included surveys from the original study were only assessed once.

### Analytic plan

All analyses were completed using SPSS 26. To characterize our sample, group differences between adolescent and child families, between those with and without food security, and between responders and non-responders at follow-up were examined using one-way analysis of variance in the case of continuous variables and chi-squared tests for categorical variables. Participant characteristics are reported for both the original sample and the follow-up sample subset; however, all analyses included only those who participated in both waves of data collection. For all analyses, baseline was the control visit from the original experiment.

Hypothesis testing was conducted using multilevel modeling because of its flexibility in terms of repeated measures factors, allowances for missing data in repeated measures designs [61], and to account for the natural interdependence that exists between parent and offspring data [62]. These models were conducted using the MIXED procedure in SPSS. All models also used a compound symmetric covariance structure. Across all models, individuals (level 1) were nested within families (level 2). The mixed procedure allows for missing values using restricted maximum likelihood estimation. Each dependent variable was checked for skew by visual examination of the histogram on the control visit. Linearity of each relationship was checked for each independent and dependent variable pair by visual examination of a scatterplots. All continuous independent variables and covariates were mean-centered prior to analysis.

The original study of this cohort established the expected baseline differences between the groups. As is typical for the developmental trajectory of DD, children had greater DD than the adolescents and their own parents and adolescents had greater DD than their own parents [49]. Further, across all age groups, food insecurity was associated with greater DD [49]. In the original study, the association between food insecurity and chronic stress only reached trend level, but food insecure parents experienced a rise in cortisol in response to a small financial loss [20]. All models in the current analysis controlled for these baseline differences and examined the associations of pandemic food insecurity, and psychological stress. Covariates for each model were chosen based on previous literature. All models controlled for number of months between assessments, participant sex, and baseline values of the dependent variable (psychological stress and DD) and baseline levels of the independent variable for each model. To examine the association of each independent variable across age groups, each model included a three-way interaction of dyad role (parent vs offspring), offspring age group (adolescent vs child), and the independent variable for each model, as well as all main effects and two-way combinations. For all models, in the case of a significant interaction, simple slope comparisons were conducted to examine the nature of the association within each group.

## Results

### Sample characterization

Seventy-seven parents consented to participate in the follow-up survey. The time between assessments (i.e., original study to follow-up survey) ranged from 4.31 to 19.59 months (M = 12.45, SD = 4.34). One parent consented to the study but did not fill out any part of the survey and their adolescent did not assent to participate. One additional parent started the survey but did not finish all measures. All parents consented to their offspring participating in the follow-up survey and 76 offspring assented to participate (37 adolescents and 39 children). One adolescent and two children started the survey after assenting but did not finish all the measures. All available data were included for all participants when possible.

Differences between families with adolescents and families with children at baseline and follow-up are presented in Table 1. Families who participated in follow-up did not significantly differ from those who did not participate in terms of offspring age, parent or offspring sex, receipt of public assistance, baseline household income per person, parent educational attainment, food security status, food security severity, perceived life stress, or delay discounting among parents or offspring (all *p* > 0.05).

### Changes over time

Within the families who participated in the follow-up survey, 62 (82%) were food secure (parent reported) at baseline. Fifty-four of those households (87%) remained food secure at follow-up and 8 (13%) became food insecure during the pandemic. Fourteen (18%) of the households with follow-up data were food insecure at baseline. Nine (64%) of those families remained food insecure at the time of follow-up and 5 (36%) became food secure during the pandemic. In terms of severity, 44 (58%) households experienced no change in food insecurity between timepoints, while 15 (20%) families reported a decrease, and 17 (22%) families reported an increase in food insecurity severity.

As expected, baseline DD was significantly correlated with follow-up DD (*r*(140) = 0.65, *p* < 0.001). As was the case in the original study and consistent with prior literature, DD was significantly correlated with participant age at follow-up *r*(147) = 0.28, *p* = *p* < 0.001). However, the number of months between assessments was not associated with the change in DD for any group. Likewise, when we created a multilevel model to predict pandemic DD, with baseline DD, time between assessments, dyad role, offspring age group, and the interaction of role, age group, and time, only baseline DD was a significant predictor of pandemic DD (*β* = 0.56, *t*(125.76) = 8.65, *p* < 0.001). This unexpected result suggests that participants with more time between assessments did not have a corresponding change in DD, regardless of the age group.

### Pandemic food insecurity and perceived life stress

**Fig. 1:**
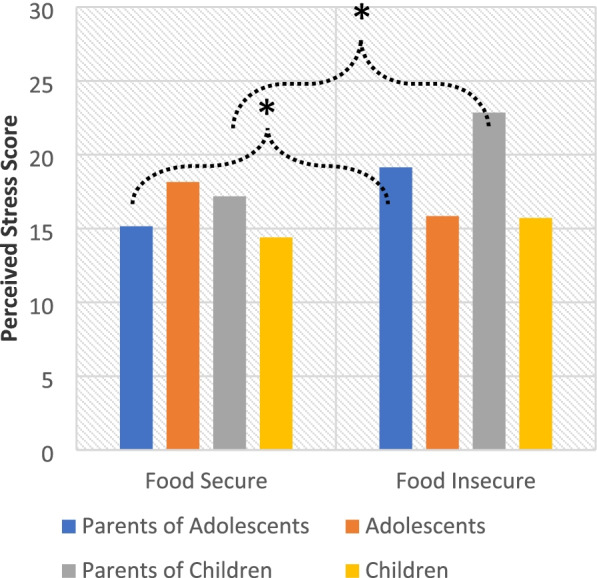
The effect of pandemic food insecurity on perceived stress. Note: Controlling for covariates and baseline levels of household food security and perceived life stress, parents who reported greater food insecurity also reported greater perceived life stress (β = -0.03, t(102.45) = -2.58, *p* = 0.011). *p < .05, **p < .01, ***p < .001

Pandemic perceived stress was associated with pandemic food insecurity (*r*(69) = 0.32, *p* = 0.007) among parents. The mixed model examining both parents and offspring showed a significant interaction of dyad role and offspring age group (*β* = -5.08, *t*(65.87) = -2.36, *p* = 0.021). Simple slopes revealed a trend for adolescents to report greater perceived life stress during the pandemic compared with the children (*β* = 3.76, *t*(117.54) = 1.91, *p* = 0.059). Finally, there was a significant interaction between pandemic food insecurity status and dyad role (*β* = 2.22, *t*(65.48) = 2.81, *p* = 0.007), with parents reporting greater pandemic food insecurity having greater pandemic perceived stress (*β* = 2.01, *t*(108.42) = 3.07, *p* = 0.003), (Fig. 1). Offspring, by contrast, did not report a change in perceived stress based on pandemic food insecurity status (*p* > 0.05).

#### Post hoc analysis

Given these results, we conducted an additional post-hoc analysis to see if parental stress was a significant predictor of offspring stress. Parent stress and offspring stress were not associated at either timepoint. This mixed model to examine this question included only offspring, used the same covariates as above, and included their parents’ ratings of perceived stress as an independent variable as well as the interaction between age group and parental stress level. Neither the interaction nor main effect were significant in this model. (*p* > 0.05).

### Pandemic food insecurity and delay discounting

Please note that DD scores are calculated from area under the curve, with higher scores indicating lower DD. Among parents, greater pandemic food insecurity was associated with greater pandemic DD (*r*(70) = -0.26, *p* = 0.029). However, this was no longer significant after controlling for baseline DD and baseline food insecurity (which were also associated with one another [49]) in the mixed model. Pandemic food insecurity also was not associated with pandemic DD among the children or adolescents (*p* > 0.05).

### Pandemic perceived life stress and delay discounting

**Fig. 2:**
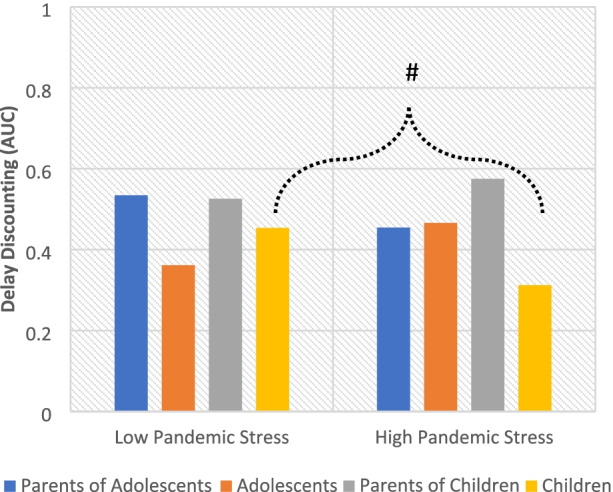
The effect of pandemic perceived stress on delay discounting. Note: Delay discounting is reversed scored, so lower numbers mean more present focus and higher numbers mean more future orientation. Controlling for covariates and baseline levels of perceived life stress and delay discounting, there was a significant interaction between dyad role, offspring age group, and pandemic perceived life stress (β = -0.03, t(102.45) = -2.58, *p* = 0.011), suggesting that there was a difference in response to stress between the age groups. Within the groups, a trend suggested that children who reported greater pandemic stress also had greater pandemic delay discounting (β = -0.01, t(118.17) = -1.83, *p* = 0.071), while parents and adolescents had no change. #*p* < .10, *p < .05, **p < .01, ***p < .001

After controlling for baseline DD and other covariates, there was a significant interaction of dyad role, offspring age group, and perceived life stress during the pandemic relative to baseline (*β* = -0.03, *t*(102.45) = -2.58, *p* = 0.011), (Fig. 2). The simple slopes for this interaction revealed a trend for the children to display slightly greater DD when their perceived life stress increased during the pandemic (*β* = -0.01, *t*(118.17) = -1.83, *p* = 0.071), while parents, adolescents, and children showed no change.

## Discussion

The current study sought to investigate how changes in food insecurity and psychological stress may be associated with one another and with changes in DD among parents, adolescents, and children, using the COVID-19 pandemic as a natural experiment. The results showed that becoming food insecure or experiencing an increase in the severity of food insecurity were associated with an increase in perceived life stress for parents, but not for children or adolescents. These changes in stress experienced by the parents did not translate into differences in DD for this group. However, children reporting greater perceived life stress during the pandemic also had greater DD during this period when compared with adolescents and parents, who did not show an association between pandemic stress and DD. These results suggest that food insecurity is particularly stressful for parents, but that DD is more sensitive to changes in stress during childhood compared with adolescence and adulthood.

Despite a strong association between food insecurity and greater DD at baseline across all age groups [49], we found that pandemic food insecurity was only associated with greater DD among parents, which was not significant after controlling for baseline DD and food insecurity. This may be because those who experienced an increase in the severity of their food insecurity had already reached a ceiling for their discounting at baseline. It is also possible that the food insecurity experienced by participants during the pandemic was more transient than is typical for this measure and participants did not experience the same narrowing of the temporal window that was observed at baseline [49] and in previous studies [63]. However, it may also be the case that food insecurity does not cause an increase in DD in the ways we have speculated in this study. Past research that has shown an association between food insecurity and DD, including the original study of this cohort, have been cross-sectional [7, 20, 64] and this association may be more complex than the current studies suggest.

At baseline, this cohort only showed a trend for an association between food insecurity and perceived life stress. The follow-up survey showed that pandemic food insecurity was particularly stressful for parents. This association was moderate, with every one-point increase on the food insecurity scaled score associated with an average 2-point increase on the perceived stress score, which ranged from 0 to 30. This finding is consistent with prior literature that has shown an association between a change in food insecurity and a change in perceived life stress [65–67]. This study furthers this finding by showing that this association is also the case when food insecurity is changing rapidly. This study also suggests that a change in household food insecurity is not associated with a change in perceived life stress in adolescents and children. Because neither stress nor food insecurity were directly manipulated by the experimenter, we cannot be sure of the causal direction between these phenomena.

We observed diverging patterns between parents, adolescents, and children in terms of what was associated with DD. While parents and adolescents showed no change, there was a trend for children to display greater DD when their pandemic stress was greater. Although the children did not report distress related to the economic burdens of the household, there are many other stressors that may have affected the children in our cohort, such as lack of social stimulation or fear of the virus. These findings are in line with previous research, which has both shown associations between greater stress and greater DD among developing youth [37, 68]. Studies have also shown that retrospectively reported childhood maltreatment and adversity are associated with differences in the biological stress pathways in the body and with greater DD and general impulsivity in adulthood [39, 40]. Longitudinally, greater exposure to stressful events has also been shown to lower self-regulatory ability in early adolescence [38]. The association in this study was quite small and only reached trend level. These findings may suggest that childhood, compared with adolescence, may be an important developmental period for psychological stress to affect DD, but more longitudinal work will be needed to answer this question.

This study is strengthened by the timing of data collection that allowed us to use the COVID-19 pandemic as a natural experiment for changes in food insecurity and stress. The sample in this study was diverse in terms of economic position and balanced for race/ethnicity across the spectrum household resources. This sample was also dyadic, which allowed for examination across family groups. However, the current results must be considered in the context of the study limitations. Although the rates of food insecurity in this study were greater than typical national estimates, there were fewer families with food insecurity than planned at baseline [49] and fewer still that reported changes in this variable, which may have limited our ability to see associations. Likewise, because the original study was stratified by offspring age group, our analyses with children and adolescents had less power than the parent analyses, limiting our ability to compare the groups. This study was also limited by the necessity to collect data virtually, particularly because we have no way of knowing the extent to which parents, siblings, or various distractions may have influenced survey responses. Finally, because the COVID-19 pandemic increased both food insecurity and stress for many families at the same time, it is difficult to establish causation between these two variables.

## Conclusions

When taken together, the current study adds to the evidence that food insecurity is associated with stress among parents. While food insecurity was not related to stress among children and adolescents, a trend in our data suggests that greater reported stress from any source may be associated with greater DD among children. This may suggest that stress, during childhood particularly, is more important in the development of self-regulation than food insecurity. Future research in this area would benefit from examining these questions with a larger sample that would allow for cross-lagged analyses of economic position, stress, and DD to better establish the directionality of these associations. Likewise, more research over a longer period is needed to understand the extent to which these factors may derail the developmental trajectory of DD and if this potential effect will persist into adulthood.

## Supplementary Information

**Additional file 1.**

## Data Availability

The datasets used and/or analyzed during the current study are available from the corresponding author on reasonable request.

## Abbreviations

DD: Delay discounting
COVID-19: Coronavirus disease 19
USDA: United States Department of Agriculture
