# Supplementary material for: The effect of food insecurity and stress on delay discounting across families: a COVID-19 natural experiment
Source: BMC Public Health. 2022 Aug 19;22:1576. doi: 10.1186/s12889-022-13969-1 (PMC9388997; doi:10.1186/s12889-022-13969-1)
Supplement: Supplementary file 1 — Additional file 1. [file 12889_2022_13969_MOESM1_ESM.docx]

Figure S1: Example of Delay Discounting Area Under the Curve

Note: The pictured values are simulated data based on real responses to show patterns of indifference points and the DD score they represent for our adult participants. Participants reported the immediate amount that was subjectively equivalent to $1000 at each delay. Delay discounting score was the area under the curve of indifference points and ranges from 0 (complete discounting of the future) to 1 (no discounting of the future) and is determined by calculating the area under the curve of indifference points over a series of delays.
